# Satisfaction with primary health care services among patients with chronic diseases: a qualitative study

**DOI:** 10.3389/fmed.2026.1650439

**Published:** 2026-01-22

**Authors:** Salah Alshagrawi, Naif Mohammed Albaqami

**Affiliations:** 1 Department of Public Health, College of Health Sciences, Saudi Electronic University, Riyadh, Saudi Arabia; 2 Family Medicine Department, Security Forces Hospital Program, Riyadh, Saudi Arabia

**Keywords:** healthcare quality, healthcare services, patient satisfaction, primary health care, quality management

## Abstract

**Background:**

Patients with chronic illnesses exhibit elevated healthcare utilization and costs, increased prevalence of depression, and impaired functional capacity. Most healthcare systems rely on primary care physicians (PCPs) to manage the treatment of patients with chronic diseases. Studies have demonstrated that patients who report high-quality care have higher treatment adherence rates, increased self-management skills, and a positive, ongoing relationship with their PCPs. This study aimed to explore the factors influencing satisfaction among patients with chronic diseases receiving primary health care (PHC) in Saudi Arabia.

**Methods:**

A qualitative technique was employed to thoroughly comprehend the intricacies of participant experiences. Online semi-structured interviews were carried out with individuals diagnosed with one or more chronic diseases and who have visited PHC. The thematic analysis framework described by Braun and Clarke was employed to code and thematically assess the data by using NVivo software. The identification of themes and sub-themes resulted in the establishment of an initial coding framework.

**Results:**

Data saturation was attained following the interviews of 46 patients (*n* = 46). The majority of participants were women, and 66% were over 50. 70% were married, 65% had college degrees, and 17% earned less than 5,000 SAR monthly. Nearly half had three or more chronic illnesses. Our thematic analysis revealed 5 themes relevant to the factors that influence the level of satisfaction among patients with chronic diseases concerning the services provided by their PCP: access to care; respect for patient-centered values, preferences, and expressed needs; coordination and integration of care; information, communication, and education; emotional and mental support; and transition and continuity.

**Conclusion:**

Our study found that dissatisfaction with accessing care remains for particularly vulnerable populations, such as the elderly, residents of remote areas, and individuals with low literacy skills. Insufficient interaction, inadequate privacy, and exclusion from the decision-making process were also significant concerns expressed by our participants, who emphasized the need for a sustained, long-term connection that fosters positive rapport, acknowledges customer competence, ensures effective communication, and addresses consumers’ mental health. Thus, it is necessary to implement fundamental modifications at various levels, including medical education, health systems, and tailored regulations, to create a general environment that promotes the establishment of an effective doctor-patient relationship.

## Introduction

In Saudi Arabia, similar to many advanced countries, around 80% of overall healthcare spending is attributed to a small fraction of the population (1, 2). A significant number of these individuals routinely utilize healthcare services for more complicated and chronic diseases (3). Chronic diseases are characterized by their prolonged duration and gradual development (4). The prevalence of chronic illnesses poses a significant challenge in nations with advanced economies as three out of every four people who are over the age of 65 throughout the world are living with various chronic illnesses (5). In Saudi Arabia, more than half of the population suffers from at least one chronic condition, and chronic diseases account for 73% of all fatalities, resulting in a significant health and financial burden (6). Patients with chronic diseases experience higher levels of healthcare use and expenses, lower self-reported health status, increased rates of depression, and diminished functional ability (7). Chronic diseases account for 84% of all healthcare spending in the US (8). In the UK, a retrospective cohort study indicated that primary care visits for patients with multiple chronic conditions accounted for 78% of all visits (9). Like many other countries, the majority of treatment and management for patients with chronic diseases in Saudi Arabia occurs within primary healthcare (PHC) settings (10).

In most healthcare systems, chronic health conditions are mainly managed by primary care physicians (PCPs) (11). In addition to directly delivering treatment involving preventive and timely intervention, PCPs serve as the gatekeepers, coordinators, and organizers of a variety of medical services for patients (12). They provide a crucial function in the management of patients with one or more chronic diseases, who necessitate various treatments to address their symptoms. Consequently, these patients typically engage in consistent and routine encounters with PCPs over an extended duration. Gaining insight into how they perceive and experience these encounters can offer valuable information about care quality (13). Research has extensively shown the importance of patient quality of care. Studies have demonstrated that patients who report high-quality care have higher rates of treatment adherence, increased self-management skills, greater motivation to manage care, and a positive, ongoing relationship with their PCPs (14). These factors are essential for effective chronic disease treatment. ‘Therefore, knowing what factors affect patient satisfaction in PHC can assist guide the provision of more suitable and efficient treatment for these patients.

One of the fundamental quality dimensions in the World Health Organization (WHO) framework for health system performance is patient satisfaction with their experience of care, which is a widely recognized promoter of patient-centered care (15, 16). Patient satisfaction with the offered healthcare services has been demonstrated to be a reliable indication of perceptions of care quality (17). Additionally, the satisfaction ratings, when contrasted with their real-life experiences of treatment, indicate patient acceptance of the provided service (18). This contrast serves as the framework by which patients evaluate the quality of care, thereby influencing their prospective standards of care (19). Thus, comprehending patients’ experiences within the context of healthcare may yield valuable insights into their perceptions, interactions, and the effects of the healthcare setting, while also identifying specific opportunities for enhancement (20, 21).

To effectively assess and address patient experiences, it is essential to understand the elements that impact these experiences within the circumstances of a healthcare environment and demographic (22). Effective patient care, particularly for patients with chronic diseases, is contingent upon the establishment of a positive relationship between patients and their PCPs (23). It has been demonstrated that how PCPs interact with patients has a significant impact on patients’ overall health (24). Patient satisfaction is significantly influenced by the communication behaviors of the PCP (25). Patient satisfaction is also specifically linked to confidence in one’s PCP, the perception of being heard, and the ability to speak with the PCP between appointments (26). Determinants of patient satisfaction with healthcare include the perception of being treated with respect, being listened to, and being regarded seriously (27). Finally, the effectiveness of communication between PCPs and their patients plays a crucial role in determining patient satisfaction, adherence to treatment plans, and overall positive health outcomes (28).

Our study was conceptually informed by the Chronic Care Model (CCM), which emphasizes productive interactions between informed, activated patients and prepared, proactive healthcare teams. The model aligns with key principles of patient-centered care and the WHO Quality Framework, highlighting essential dimensions such as access, coordination, communication, and continuity. These concepts provided the analytical lens for exploring how patients with chronic diseases experience and evaluate primary health care services in Saudi Arabia.

Although it is crucial to understand the satisfaction of patients with chronic diseases with the care received in PHC, research is scarce in Saudi Arabia about such issues (29). Although there are some studies that examined patient satisfaction with healthcare services in Saudi Arabia, the majority of such studies have adopted quantitative data collection methods, other studies focused on the general population instead of patients with particular conditions (30). Thus, there is still a need to fully understand how patients with chronic diseases experience and perceive the service provided by PHC. Current research has not adequality reflected the complex, real-life experiences of these patients, especially concerning communication, access, and continuity of care. Therefore, the present study aimed to explore the factors influencing patient satisfaction with primary health care (PHC) services among individuals living with chronic diseases in Saudi Arabia. Using a qualitative approach, this study sought to gain an in-depth understanding of patients’ experiences and perceptions of care delivered by primary care physicians.

## Methods

### Study design

A qualitative approach was implemented to fully understand and demonstrate the scope and depth of participant experiences (31). Online semi-structured interviews were carried out with individuals diagnosed with one or more chronic diseases. This method was chosen to prioritize participant safety and facilitate accessibility throughout the data collection process. We acknowledge that conducting interviews online may result in limited participation from individuals with lower socioeconomic backgrounds or those with restricted digital literacy, who may feel less at ease using online platforms.

### Research team and reflexivity

The interviews were conducted by the first author (SA), who holds a background in public health and qualitative research. At the time of the study, he had no professional or personal relationship with the participants. The researcher’s prior experience in healthcare may have influenced the interpretation of participants’ responses; therefore, several measures were taken to mitigate potential bias. These included maintaining a reflexive journal throughout the data collection and analysis process, regularly discussing interpretations with the co-researcher (NA), and jointly reviewing coded transcripts to ensure that emerging themes accurately reflected the participants’ perspectives rather than the researchers’ preconceptions. This reflexive approach enhanced the credibility and trustworthiness of the findings.

### Participants

The participants were recruited from a PHC center located within a big hospital in Riyadh, Saudi Arabia. This research was carried out at a singular primary health care center in Riyadh, chosen for its ability to serve to a varied patient demographic with multiple chronic conditions and for offering extensive primary health care services that reflect standard practices within Saudi Arabia’s health care system. The single-site design facilitated comprehensive engagement with participants and ensured uniformity in data collection procedures. The study prioritized depth of understanding over breadth of coverage, reflecting its qualitative and exploratory nature. Future research may extend to various centers to analyze differences across regions and types of facilities. A purposive sampling strategy was employed to ensure variation in age, gender, and type of chronic disease. Eligible patients were identified from hospital records, and invitations were sent via email to those who met the inclusion criteria. Participants were recruited until data saturation was achieved. Eligible participants were identified from the hospital’s primary health care center records in collaboration with the administrative office. Each eligible patient received a personalized email invitation from the research team explaining the purpose of the study, inclusion criteria, and the voluntary nature of participation. The email included an information sheet and contact details for further inquiries. Interested participants were invited to reply directly to the researcher to schedule an interview at their convenience. All correspondence and recruitment materials were approved by the hospital’s ethics committee to ensure confidentiality and compliance with institutional regulations. From the pool of eligible patients identified in the hospital records, a subset of patients was purposively invited to participate to ensure variation in age, gender, and type of chronic condition. Data saturation was attained following the interviews of 46 patients (*n* = 46). Patients included in the study had a wide range of backgrounds and attributes, including age, various years of previous experience, and the existence of a rare or unknown ailment. Every participant finished their interviews, and there were no instances of repeat interviews. The average duration of interviews was 65 min, with a minimum of 36 min and a maximum of 75 min.

We targeted individuals with one or more chronic medical diseases, characterized as those lasting 6 months or more following diagnosis or identification of a long-term condition (10).

The inclusion criteria for participants were as follows:

- Having one or more chronic conditions with symptoms lasting ≥6 months (regardless of having an official medical diagnosis or not).
- Currently seeing a PCP for the management of the condition(s).
- Minimum age of 18 years.
- Has no cognitive, psychological, or other limitations that would prohibit them from taking part in the study on their own.

Based on previous comparable studies (13, 32), we sought to recruit at least 25 people to guarantee the representativeness of the sample. The sampling strategy was used to complete the recruitment of patients until thematic saturation.

### Data collection

Between January and April 2023, we conducted qualitative, semi-structured interviews, each lasting roughly 63–75 min. Prior to participation, all eligible participants received an information sheet detailing the study objectives, procedures, risks, and confidentiality protections, and provided written informed consent electronically before the interviews were conducted. The researcher (SA) conducting the interviews possessed a strong background in interview techniques and had no professional relationship with the participants. Before data collection, the semi-structured interview guide was piloted with two participants who met the inclusion criteria but were not included in the final sample. Feedback from these pilot interviews was used to refine question wording and flow to ensure clarity and cultural appropriateness. To protect the participants’ anonymity and allow them to freely express themselves without feeling any social pressure, all the interviews were conducted online (33). There were no repeated or follow-up interviews in which the participants were involved. The semi-structured interview guides were adopted to collect (i) exhaustive data on patient satisfaction and experience, encompassing a comprehensive overview of a standard consultation from setting up an appointment to departure from the office, along with occurrences that transpired between visits (ii) participants’ viewpoints about critical elements of patient experience and quality enhancement in managing chronic diseases by PCPs in the PHC practice. All interviews were audio-recorded with participants’ permission and transcribed verbatim. Transcription was undertaken by the research team, and all transcripts were reviewed against the audio recordings for accuracy prior to analysis. Identifying information was removed during transcription to ensure anonymity.”

Topics and questions for discussion were aligned with the Chronic Care Model (CCM) (34, 35). The CCM offers a structured approach to the essential elements required for effective integrated chronic disease management (Table 1). It encompasses the functions of the health system and healthcare providers, policy, the community, and patients. Participants’ self-reported education level and monthly income were utilized to capture socioeconomic factors, as these are widely acknowledged as significant indicators of socioeconomic status. A composite socioeconomic level was not developed due to the qualitative focus of this study, which sought to illustrate contextual diversity rather than measure associations. The distinct reporting of income and education facilitated a more detailed analysis of how each element affected patients’ access, communication, and satisfaction in primary health care services. All participants were informed of the study’s overarching goals, which included investigating the patients with chronic diseases’ satisfaction of the provided PCP’s care. The interviews were recorded in audio format and transcribed word for word. During this procedure, all data were anonymized to ensure anonymity through the use of distinctive codes. Interviews were conducted until theoretical saturation was reached. The interview guide (Table 1) concentrated on patients’ experiences and satisfaction regarding consultations with primary care physicians (PCPs) in primary health care centers. Participants were requested to consider their latest interactions with primary care providers, as opposed to those involving hospital-based or specialty care. It is important to note that numerous patients with chronic conditions often engage with various healthcare providers, which may have impacted their responses based on their overall healthcare experiences. These instances were regarded as integral to the patient’s comprehensive understanding of care continuity throughout the various levels of the system.

**Table 1:** Interview questions and prompts.

Questions and prompts
1. Could you walk me through a normal visit to your PCP, from making an appointment to leaving the office? How easy or difficult is it to book an appointment with the clinic? How easy or difficult is it to travel to the clinic? How friendly are the reception staff at the clinic? Does your PCP follow-up with you after visits? OR Does your PCP or practice nurse check up on you from time to time (at home/by phone)? 2. In general, how well do you get along with your PCP? How long have you been seeing this PCP? How often do you visit him/her in a year? What conditions are you currently seeing your PCP for? How long have you had [this condition]? Does your PCP know you well as a person? Is your PCP easy to understand most of the time? Do they explain things carefully? Do you often make decisions about your health together with your PCP? Is that important to you? How well do they listen to any worries or concerns you might have? 3. Based on your experiences of care at the PHC clinic, what are the most important areas for improvement? Does your PCP know about these areas of concern? How comfortable would you feel about talking to your PCP about these areas of concern? Does your PCP or other staff at the clinic ask you for your feedback on your experience of care? If yes, how do they ask you for your feedback? If no, is this something you would find useful or important to do regularly?

### Data analysis

Data were analyzed using Braun and Clarke’s (35) six-phase approach to thematic analysis, which involves familiarization with the data, generating initial codes, searching for themes, reviewing themes, defining and naming themes, and producing the report. Two researchers (SA and NA) independently coded an initial subset of transcripts, then met regularly to discuss and compare coding decisions (36). Discrepancies were resolved through consensus discussions, and the coding framework was refined iteratively until agreement was achieved. This collaborative process enhanced the credibility and dependability of the analysis. Following the preliminary examination and acclimatization to the transcripts. Inductive coding was performed on a preliminary set of transcripts. The identification of themes and sub-themes led to the completion of an initial coding framework. The analysis of data was conducted collaboratively by the two researchers (SA, NA), with continuous discussions on themes and iterative revisions of coding guiding the process. During this process, the researchers engaged in discussions regarding disagreements and variations until a consensus was reached. The analysis and inductive coding scheme were conducted utilizing NVIVO 11. (NVivo qualitative data analysis Software; QSR International Pty Ltd.).

### Ethical considerations

This study was approved by a Human Research Ethics Committee at the Security Forces Hospital (IRB number: 22-577-13). The study was conducted in accordance with the principles of the Declaration of Helsinki. All participants received a participant information sheet and supplied their written informed consent to engage in the study. The data was maintained in confidentiality and utilized solely for the objectives of this study.

### Trustworthiness of the study

To ensure the trustworthiness of this qualitative study, we followed the criteria proposed by Lincoln and Guba (37), addressing credibility, transferability, dependability, and confirmability. Credibility was enhanced through prolonged engagement with the data, researcher triangulation (two researchers independently coded and interpreted transcripts), and regular consensus discussions. Transferability was supported by providing detailed descriptions of the study setting, participants, and context to allow readers to determine applicability to other settings. Dependability was maintained through a clear audit trail documenting all methodological decisions and data analysis steps. Confirmability was strengthened by maintaining a reflexive journal and conducting peer debriefing to minimize researcher bias and ensure that findings reflected participants’ perspectives rather than researchers’ assumptions.

## Results

### Characteristics of participants

More than half of the participants (*n* = 46) were female, and approximately 66% were aged 50 or above. A total of 70% were married, 65% held an undergraduate degree or above, and 17% earned less than 5,000 SAR per month. Nearly half of the participants endured three or more chronic diseases. Participants frequently switched their primary care providers, with patients indicating a median duration of 6 months spent with the same PCP. Table 2 outlines the characteristics of participants. We investigated the impact of participants’ demographic characteristics, such as age, gender, education level, and the number of chronic diseases, on their impressions and the themes that arose during thematic analysis. No systematic variations were observed in the overall pattern of responses. Nevertheless, some distinctions were observed. Older individuals often discussed challenges related to accessing care and mobility, while female participants focused more on their comfort with same-gender doctors and concerns regarding privacy.

**Table 2:** Participants’ demographic characteristics (*n* = 46).

Characteristic	Description	*n* (%)
Sex	Female	25 (54.3)
	Male	21 (45.7)
Marital status	Married	32 (69.6)
	Single	11 (23.9)
	Divorced	2 (4.4)
	Widow	1 (2.1)
Age	18–30	1 (2.1)
	31–49	8 (17.4)
	50–64	19 (41.3)
	60 and above	18 (39.1)
Education level	Postgraduate education	4 (8.7)
	Undergraduate education	26 (56.5)
	Primary/secondary school	13 (28.3)
	No general education	3 (6.5)
Income level	More than 15,000 (SAR/month)	6 (13)
	10,000–15,000 (SAR/month)	15 (32.6)
	5,000–9,999 (SAR/month)	17 (37)
	Less than 5,000 (SAR/month)	8 (17.4)
Number of chronic conditions	One	5 (10.8)
	Two	17 (36.9)
	Three	16 (34.8)
	More than 3	8 (17.5)
Median years lived with condition(s) (range)	9 (1–21) years	
Median years seeing current GP (range)	6 months (2 months–1 year)	
Total PCP contacts in the past 6 months (range)	5 (1–8)	

PCP, primary care provider.

Our thematic analysis revealed 5 themes relevant to the factors that influence the level of satisfaction among patients with chronic diseases concerning the services provided by their PCP: access to care; respect for patient-centered values, preferences, and expressed needs; coordination and integration of care; information, communication, and education; emotional and mental support; and transition and continuity (Table 3).

**Table 3:** Summary of themes and subthemes identified from thematic analysis.

Main theme	Subthemes	Description
Access to care	– Physical accessibility and mobility challenges – Appointment scheduling and waiting times	Barriers related to distance, mobility, and booking systems
Information, communication, and education	– Clarity of explanations – Health literacy and patient education – Autonomy and shared decision-making – Privacy and gender preferences	Quality of verbal and written communication; patient understanding
Emotional and mental support	– Psychological well-being – Empathy and compassion	Addressing mental health and emotional aspects of chronic illness
Coordination and integration of care	– Communication among providers – Continuity across services	Cooperation between PHC and specialists; timely feedback on test results
Transition and continuity	– Long-term relationships – Consistency of follow-up	Sustained engagement with the same PCP to ensure effective chronic care

### Access to care

While some participants reported no significant difficulties obtaining PHC, we noted that barriers remained for specific vulnerable groups, including the elderly, those residing in remote regions, and individuals with low literacy levels.

For instance, an elderly participant indicated that he depended on family assistance to arrange an appointment with a PCP: “*Setting up an appointment is never easy for me. That’s why I have to ask my son to make an appointment for me to get my cholesterol checked or when I have a strange headache.*” (P-15).

Several participants mentioned physical mobility as a determinant of their capacity to access the PHC center, with chronic discomfort, exhaustion, and physical impairments being among the difficulties that were mentioned.

One participant illustrated this point: “*think about it twice before I go to the doctor. It’s hard for me to get around and get to the hospital. Because my knees hurt and I get short of breath, it is tiring and stressful for me.*” (P-35).

Scheduling visits was crucial for providing continuous evaluation, treatment, and monitoring. Regular scheduling appointments were not perceived to present a significant challenge for participants who had a regular PCP. Some participants highlighted some difficulties in scheduling at the beginning of the available self-booking options via phone or online: “*In the beginning, it was hard for me to use the phone to make a self-booking appointment. But, I learned how to do it over time, which made setting up follow-up visits even easier than the old way of making appointments.*” (P-8).

Other participants preferred the self-booking system but suggested having mobile messages and call reminders to ensure adherence to their appointments. Participants living in urban areas faced several challenges when trying to get PHC, including a lack of PCP in the area, long travel times to get to a medical center, and inconsistent provider availability and business hours.

“*Living outside of the city has made it more difficult for me to attend my appointments, particularly when considering the amount of time and effort that is required to go to the clinic as well as the limited number of hours that the clinic is open for service.*” (P-21).

A few participants mentioned that their PHC or PCP was accommodating regarding communication and appointment scheduling, allowing flexibility in the provider’s schedule for potential walk-ins, even for those without pre-booked appointments. Additionally, our participants experienced a wide range of waiting times for services at PHC centers. Despite the fact that some participants expressed frustration with what they considered to be excessive delays, none of them identified this as a significant obstacle to seeing their PCP.

“… *wait time will* var*y on the day of the week and the doctor you are seeing, but on average, I am happy with the time I have to wait to see my doctor. It’s possible that a longer wait time means my doctor is good and worth the wait.*” (P-27).

Participants who are more likely to be vulnerable, such as those with more serious illnesses or co-morbidities connected to mental illness, were more concerned about the waiting space. The waiting area’s suitability for these participants was negatively affected by noise, crowding, and a lack of privacy.

### Information, communication, and education

This theme focuses on the extent to which PCPs respect patients’ individuality, autonomy, privacy, cultural or gender preferences, how information was conveyed, the clarity of explanations, and the extent to which education and guidance supported patients’ understanding and self-management. It captures how patients felt heard, valued, and involved in decision-making about their own care. Social and cultural challenges to meeting patient requirements, such as the availability of culturally compatible providers and gender-based preferences for PCPs, were among the issues that were raised by the participants.

An elderly participant stated: “*Finding a doctor who understands your health problem is hard these days. A young doctor I had once made me feel like he didn’t have enough experience by listening to what I had to say about my problem. I want to talk to an old doctor about my life experiences.*” (P-34).

A female participant mentioned: “*There is a significant difference between my appointment with a man and a female doctor. I feel more at ease when I express my concerns with a female doctor. Sometimes I had to reschedule my appointment because of the inconvenience before meeting a male doctor.*” (P-26).

Participants facing mental health challenges expressed additional concerns. Due to cultural and societal stigma and fear of disclosure of personal information, some patients expressed reluctance to disclose their mental health difficulties.

“*not sure if I’m ready to tell my doctor about my mental problem. This may make them think about me differently. My problems could also be talked about with the staff. I don’t trust people or what they say about me.*” (P-4)

A participant shared an example illustrating the effects of her inadequate health literacy: “*The thing that bothers me is when the doctor talks about my problem in medical words that I can understand. There are times when it is too hard to understand, even when I ask for help. That’s why I have to look up online what the doctor said after the visit.*” (P-31).

Ensuring privacy is essential for patients to be satisfied with their treatment. Multiple participants have expressed their worries over their privacy as a result of the testing, exams, and medical care they have received.

“*At different points during the visit, you will talk to different staff members and they sometimes ask about personal things. It scares me to talk about private things with people I don't know well or who will talk about me next.*” (P-16).

Privacy also presented an issue in the waiting room and registration area, wherein a patient may expose critical details to individuals who are not engaged in the care they received or be listened to by other patients who are waiting.

*“I am annoyed standing in front of a nurse or receptionist and being expected to explain to them what I want to see the doctor about while everybody in the waiting room is listening.”* (P-30).

Participants in this study had high expectations that PCPs would value and consider their opinions. In many cases, patients reported feeling confident in their knowledge and having a high level of health literacy. They believed it was essential for PCPs to recognize and validate their competence, particularly as collaborators in the decision-making process.

“*When I go to my appointment, I generally know a lot about my problem. I want the doctor to listen to me carefully and tell me what he or she thinks about my health problems and treatment plan so that we can both choose the best way to treat my condition.*” (P-44).

A significant number of individuals indicated a preference for clinicians who refrained from imposing their priorities, choosing instead to honor the autonomy of consumers in making the ultimate decisions regarding their care. Involvement in treatment decisions was a crucial aspect of collaboration that intersects with concepts of compassion, accessibility, and overall perspectives on the wellness of patients:

“*When my doctor talks to me, it sounds like I’m in charge.” He suggests different treatment plans and then asks me what I think. I like having these kinds of talks with my doctor. He didn’t tell me I had to do it or scare me. He gives me some choices to think about and make my own choices.*” (P-3).

Certain participants perceived that they previously had limited influence over their prescription medications or the availability of alternative options: “*The doctor only asked a few questions and then wrote a prescription without giving any more information.*” (P-18).

Involvement in medication decisions and exploration of alternative and complementary therapies were two occasions that participants mentioned: “*I am curious as to why they do not propose alternative treatments that do not include the use of medicines. In my opinion, the only thing that they recommend is the use of chemicals. What about using natural healing methods or remedies at home?”* (P-13).

Participants sought complete answers to their examinations or laboratory tests and frequently desired additional data than is usually offered.

*“Don’t tell me something just because you believe I should know. I’d like to know what the test was for. I’d like to know what you were looking for and what the findings were. I’d like to track my iron and sugar levels and compare this year’s results to last year’s. This allows you to better understand your health status*.” (P-2).

Participants reported a positive rapport with their PCP as very important to their overall satisfaction in PHC practice and to the success of their therapeutic relationship. The attributes that participants valued the most were their friendliness and professionalism, which made conversations and interactions with others more comfortable: *“The way the doctor asks about my condition, listens, and interacts with me shows that she cares. She seems friendly and professional at the same time, which makes me more relaxed and at ease when I talk about my condition.”*(P-19).

Participants stated that seeing a PCP who was familiar with them made them feel more at ease because of the accumulated trust and the eliminated need to describe their illness and complaints during each appointment.

“*I’ve been going to the same doctor for years.” He trusts me, and I trust him too. Besides that, he knows a lot about my illness and everything that happened because of it. When I go to any meeting with him, I’m happy.*” (P-41).

Participants regarded effective communication as the foundation of the therapeutic relationship. The importance of open communication with one’s PCP extends beyond simply exchanging medical records; it also includes patients’ ability to ask questions, voice concerns, and receive attentive, nonjudgmental responses. Participants also mentioned that PCP must offer more time to listen and being able to disagree with their doctor’s decision without feeling judged or criticized is an important aspect of excellent communication in a clinical setting: *“I am comfortable with disagreeing with him in case his decision is not suitable for me. And I do not feel threatened or judged by him for refusing to follow his recommendations. He listens to my concerns and to me this is the most important thing in my treatment plan.”* (P-7).

An additional component of effective communication was non-verbal clues and interpreting body language, which the PCP had a good enough familiarity with their patients to do even when their patients were unable to speak for themselves: *“… at times she can understand what I feel by my expression and body language. So, she asked whether I am concerned about something she said or whether I understood her point.”* (P-32).

The PCPs’ capacity for active listening also seemed to support effective communication. Participants defined this as paying close attention to what patients have to say and then reassuring them of their worries and anxieties. Patients, especially patients with mental health problems, perceived this technique as beneficial in mitigating worries and anxiety, which they regarded as a crucial component of their responsibilities as caregivers.

*“… she listens. I can tell that she cares because she never interrupts me while I’m talking. She is a good listener and that is what makes me feel comfortable talking to her.”* (P-13).

### Emotional and mental support

Some participants in our study reported challenges associated with their mental health, asserting that these issues significantly affected the whole treatment process. Several participants identified mental health issues as a substantial challenge stemming from the complexities of managing a chronic disease. As part of their usual care, they reported that they expected their PCP to inquire about their mental health.

One participant described her PCP’s caring attitude: “*Apart from controlling my physical problems, she also makes sure she is always following my mental health state of affairs. She constantly gives my mental health a top priority and shows a great degree of sensitivity.”* (P-45).

Taking care of the patient’s mental health was seen by some participants as an indication of the PCP’s skill, including their capacity to be perceptive and comprehensive.

*“When I go to her, she takes care of more than just the illness I came in. She is going to ask me about my emotional and psychological well-being.”* (P-7).

Another participant provided a rather negative opinion: “*Mental health is something that is not looked after by my doctor. His focus is only on the physical part, the signs and symptoms. What about my whole health and wellness, my emotional and mental health? This might be the reason for all of my diseases.”* (P-38).

### Coordination and integration of care

The patient’s health care process became more complex due to the requirement for numerous laboratory tests and referrals, as well as the seriousness of some test results. Participants voiced concerns that communication between providers may suffer as a result of fragmented healthcare. Due to numerous patients being frequently referred to other providers for various elements of their care and testing, the importance of open communication channels between patients and specialists, as well as between specialists and primary care physicians, was underscored: “*seems like each doctor or nurse has their own idea, which can make you feel lost. Also, they don’t seem to be getting the information they need from each other. They use questions that are already in the system and ask what other doctors said instead of having a direct way for them to talk to each other and share information.*” (P-16).

The integration of care was also assessed by determining whether both favorable and undesirable test findings were delivered promptly, and privately, and offered to the patient with information and discussion.

*“I wish I could get lab reports faster and with less trouble. Also, it would be beneficial to have a lengthy discussion with the doctor when the results are made public, rather than asking family, friends, and the internet what the results mean.”* (P-27).

In addition, patients expressed a need for a wider variety of care options, including counseling and various forms of education. This encompassed parenting, sexual functioning, abuse, reproductive health, and menopause, as well as practical matters like medicine, diet, nutrition, disease prevention, and treatment.

*“doesn’t my doctor offer different things other than prescribing medications? What about recommending a well-studied lifestyle, diet plan, or an education workshop? Wellness isn’t just about our bodies; it’s also about our minds and souls.”* (P-42).

As a result of receiving conflicting information from different doctors over the course of time or from multiple medical professionals, participants voiced their disappointment that decision-making became increasingly challenging for them under these circumstances.

*“I have been dealing with diabetes for more than 10 years and I can tell you with certainty that I have trouble making a proper decision because of the different feedback I received from my doctors. If you don’t trust your doctors, who should you trust.”* (P-25).

Participants expressed concerns about the exchange of information among clinicians across various health services as well, citing conflicting messages as a significant issue.

*“When I’m referred to a new doctor, I’m terrified of starting over with another treatment plan and side effects.”* (P-37).

### Transition and continuity

The significance of maintaining a consistent, long-term PCP in the management of their conditions was expressed by most participants who emphasized the importance of maintaining regular visits to their PCP.

“*Seeing my doctor regularly is an important part of managing my diabetes well because it lets them check up on my care plans and keep an eye on any problems that might arise.*” (P-13).

Participants believed that this was an essential component of chronic care because it allowed their primary care physician to collect essential, relevant knowledge about them during the course of their treatment. This knowledge included a full assessment of the individual’s medical condition, shifting signs and symptoms, and physical and emotional needs.

Unfortunately, not all participants were eager to keep their scheduled visits. Some participants reported that they just did not have the time or preferred to use PHC for more urgent issues.

“*I made an effort to maintain the regularity of my visits to the clinic because I am aware that this will be beneficial to me in the long run. On the other hand, life tends to get in the way and become distracting.*” (P-24).

## Discussion

This study sought to address a significant knowledge gap in understanding the satisfaction of PHC services among patients with chronic diseases. It aims, through qualitative research, to explore patients’ satisfaction through their experience with access to care, including their needs, barriers, and potential areas for enhancement in health services. We found that patient satisfaction was mainly influenced by 5 main broad categories: access to care; respect for patient-centered values, preferences, and expressed needs; coordination and integration of care; information, communication, and education; emotional support; transition and continuity.

### Access to care

Overall, our participants did not report any significant impediments to care. This was corroborated by other researchers. Several studies examining patients with chronic diseases reported overall positive experiences with access to healthcare services. Patients exhibited heightened confidence due to the availability of timely and accessible services (38). In our study, however, major barriers to accessing care were highlighted by specific groups, including the elderly, residents of remote areas, and individuals with low understanding of medical terms. Patients’ capacity to reach or physically access services was the most salient and recurrent difficulty in accessing PHC. Additional research has demonstrated comparable findings and offered justifications including physical mobility limitations, chronic exhaustion, and discomfort that hindered access to basic care (39). Other studies identified additional factors, including anxiety, forgetfulness, multimorbidity, and impaired vision and/or hearing (40). Such barriers to accessibility can be overcome by providing alternative service delivery methods, such as over-the-phone or online consultations. Additionally, healthcare organizations can ensure the availability of PCP home visits for certain demographics that are vulnerable and unable to attend physical appointments. Since none of our participants have experience with home visits, future studies could explore patients’ preferences between home visits and consultations conducted over the phone or online.

Our study found that even though participants first encountered challenges with the self-booking system, they were satisfied with how simple it was to make appointments. Recent studies indicated that self-booking systems can decrease no-show rates and waiting times, enhance convenience for patients, and potentially boost provider efficiency by alleviating the workload associated with managing appointments (41). However, self-booking has also elicited concerns about equitable access, patient safety, the management of scheduling complexities, and physicians’ autonomy over their work schedules (42). Thus, developing better and safer self-booking systems is essential to help PHC practices match patient needs and available resources.

### Information, communication, and education

In our study, female patients demonstrated a preference for attending appointments with female physicians rather than male physicians. This has also been demonstrated in other studies (43). This may be because the female patient required additional time to discuss her concerns. A meta-analysis indicated that female physicians are generally more patient-centered and have significantly longer visits with their patients, averaging 2 min longer in duration compared to their male counterparts (44). In the Saudi PHC context, where cultural norms emphasise modesty and gender-segregated interactions, this preference may carry added weight, influencing how openly patients communicate and engage in their care (43).

We also found that older patients in our study prefer physicians who are older and can understand their needs. The interaction between older patients and their physicians significantly impacts satisfaction, adherence, and various health-related outcomes. The relationship between a physician and a patient can serve as a form of therapy for the patient, especially for older individuals facing comorbid conditions, depression, intricate treatment plans, and restricted resources (45). Therefore, initiatives and programs can be established to enhance physicians’ attention, warmth, care, concern, and practical support, which can significantly impact the health of older patients.

Another significant finding for utilizing healthcare involves privacy. Some participants experienced a sense of diminished privacy in public spaces as they were compelled to disclose personal details to individuals other than their healthcare provider or became at risk of having their conversations listened to by other people. A systematic review identified concerns of patients regarding the diminished level of privacy that is associated with the utilization of pharmacies and the lack of the ability to have private conversations (46). Consequently, PCP and other providers must exercise professional judgment in utilizing private spaces and methods to ensure privacy, while also being attuned to patients’ needs. Staff training should also incorporate specific communication skills to enhance privacy, such as lowering voice levels. Our theme of privacy and stigma is rooted not only in individual attitudes but also in systemic factors such as how PHC services in Saudi Arabia are organized, how sensitive information is handled, and how sociocultural stigma about chronic illness and mental health may suppress patient voice. For example, research has highlighted how cultural values around reputation and disclosure in Saudi society limit open discussion of health conditions (47).

Participants in this study held strong expectations that PCPs would involve them in decisions, respect their autonomy, and take into account their opinions. Based on the findings of various research, patients value the experience of being recognized for their knowledge and viewpoints. Despite the evidence of the benefits and significance of patient involvement in managing their medical conditions, substantial obstacles to patient participation in practice still exist (48). Significant obstacles for patients were linked to their health status, frequently associated with age, as well as the necessity for patients to be empowered in managing their health conditions and diagnoses, along with support to tackle their lack of health literacy (49). Thus, the duty of healthcare providers is crucial in empowering patients during the engagement process by facilitating clinician-patient communication, encouraging inquiries, promoting participation in specific acts, and providing positive reinforcement and support.

Although communication and respect are interrelated components of patient-centered care, they were treated as separate themes to highlight distinct aspects of the patient–provider relationship. “Respect for patient-centered values” reflects patients’ perceptions of autonomy, privacy, and involvement in care decisions, whereas “information, communication, and education” captures the functional and informational processes that enable understanding and engagement. In our study, participants appreciated a positive relationship with their PCP, characterized by traits like friendliness and professionalism, which facilitated comfortable conversations and interactions in a judgment-free atmosphere. Our findings align with previous research highlighting the significance of developing provider-patient relationships that transcend biomedical methodologies in the management of chronic diseases (50). Research on patients dealing with long-term health issues, such as obesity or mental illness, has also highlighted the significance of providers actively listening to their patients and making an effort to impact their level of satisfaction (51). Nevertheless, Participants in our study reported that PCP must allocate additional time for them to pose inquiries and voice concerns. Attentively listening to patients and sustaining an active communication channel necessitates that clinicians allocate time to every patient. In primary care, however, time limits are a barrier, particularly for patients who are dealing with chronic diseases (52). Innovative institutional strategies are required to enable providers to dedicate additional time to patients with chronic illnesses (53).

### Emotional and mental support

Some participants in our study expected that their PCP would ask questions about and demonstrate interest in their mental health. Other research has found that people with chronic illnesses need to have conversations regarding the social, cultural, spiritual, and emotional challenges they face on a daily basis (50). There is substantial evidence in the literature linking persistent physical sickness to mood disorders like depression and anxiety (54). The relationship is reciprocal as compared to the general population, those with long-term physical illnesses are more likely to suffer from sadness and anxiety (54). Consequently, mental illness worsens the functional consequences, quality of life, and increased mortality that come with chronic diseases (55). Mental health services, such as evaluation, planning, and review in primary care settings, should be a part of chronic disease management programs to help patients cope with this stress and achieve better results (56). Additionally, maintaining consistent mental health care may be a crucial strategy for meeting customer demands for comprehensive, person-centered chronic care. The work that has been done in the past has identified quite distinct models that patients and physicians use to comprehend sickness. Patients are concerned with the effect that the disease has on their lives, whereas physicians are concerned with the pathophysiological abnormalities that impact patients’ physical well-being (57, 58). However, patients have the perception that they are receiving considerably better care when PCPs pay attention to the urgent issues that are affecting their lives and demonstrate care and respect for them and there is a growing body of research indicating that they achieve superior health results (59). PCPs have attributed the lack of attention to patient needs to the limited amount of time available for consultations (60). In one study, physicians indicated diminished rapport linked to reduced average consultation time (61). However, another study contradicted this by demonstrating that the duration of consultation is an inadequate indicator of quality when the true requirement is for high engagement (62). More research is needed to examine the relationship between consultation time and health outcomes in primary care settings.

The focus that participants placed on emotional and psychological well-being highlights the significance of incorporating mental health into primary care delivery. This is in accordance with worldwide demands for cohesive primary health care, where both physical and mental health are addressed collaboratively by diverse teams of professionals (52). In the context of Saudi Arabia, the findings underscore an opportunity to enhance the capacity of PHC centers to deliver essential mental health screening, counseling, and efficient referral pathways. Integrating mental health into primary healthcare aligns with the Saudi Vision 2030 health transformation program, emphasizing a holistic, patient-centered, and preventive approach to care (63). Initiatives at the policy level may involve training primary care providers in effective mental health communication, integrating mental health professionals into primary health care centers, and creating referral and follow-up systems that guarantee continuity between physical and psychological care.

### Coordination and integration of care

The perceived absence of integration and coordination among healthcare providers was a source of concern for the participants in our study. This was marked by insufficient exchange of information between the physicians and delayed responses to testing and laboratory results. Care coordination is a process that guarantees that all providers and organizations involved in health care provide the appropriate care at the appropriate moment. The management of chronic diseases, specifically chronic obstructive pulmonary disease and diabetes, demonstrated consistency in clinical oversight, including the allocation of care across several levels and expedited access and referral to mitigate future hospitalizations and long-term complications associated with diabetes (64, 65). Patients were safer and had better outcomes as a result of interprofessional collaborative care, which guaranteed coordinated care addressing foreseeable difficulties (66, 67). Various factors can impact the success of multidisciplinary care coordination strategies. These include interpersonal factors, such as language barriers, familiarity, trust, and respect; professional factors, such as individual qualifications and incentives; internal factors, such as structure and composition; and a shared mission (68). Current health systems and care models prioritize primary care on sickness treatment, with insufficient emphasis on health promotion and prevention. Disease-centric models fail to fulfill the health requirements of individuals and communities; rather, they necessitate person-centered integrated health care to accommodate evolving epidemiological and demographic changes (69). Moreover, technological advancements can serve as catalysts for altering primary care to access those who lack access (70, 71).

### Transition and continuity

The majority of participants acknowledged the importance of sustaining consistent, long-term care with their PCP in the management of their conditions. While some patients maintained the same PCP or healthcare provider for some time, others shifted between several doctors and providers. These changes stemmed from changes in patient preferences, turnover among healthcare providers, or the voluntary pursuit of alternative providers for superior care (72, 73). Research has shown that patients who see the same PCP over the years report higher levels of trust, which in turn improves medical rapport and interpersonal communication (74). The continuity of care enhances physicians’ ability to execute clinical activities more efficiently, hence enhancing the diagnosis and management of chronic and complex illnesses (75). Additionally, care continuity allows PCPs to gain a better understanding of the patients they treat as individuals with complex needs, which is frequently regarded as essential for the provision of patient-centered chronic care (50). Patients in Saudi Arabia are not obligated to remain with the same PCP. Individuals can switch their PCP easily if they feel their care is lacking. Thus, to ensure that patients return, the PCP has to make an effort to negotiate with them and manage their expectations.

### Implications for primary care practice

Our findings indicate that primary care physicians ought to emphasize effective communication, empathy, and shared decision-making as essential elements of patient satisfaction. It is essential to dedicate time for active listening, guarantee that medical explanations are clear and culturally relevant, and engage patients in the treatment planning process. Furthermore, primary care providers should enhance their understanding of patient enablement and empowerment principles, promoting an active role for patients in managing their chronic conditions.

At the system level, primary care management should facilitate this transformation by offering ongoing development in patient-centered communication, empathy, and mental health awareness. Establishing consistent feedback mechanisms and mentorship initiatives can effectively strengthen these competencies in everyday practice. Collectively, these strategies can facilitate the transition of primary healthcare in Saudi Arabia towards a more person-centered model that aligns with international.

### Broaden interpretation

Although earlier research in Saudi Arabia has measured patient satisfaction, there has been limited exploration of the real-life experiences that inform these perceptions. Our findings extend past mere satisfaction scores by uncovering the ways in which cultural expectations, gender norms, and communication styles influence patients’ feelings of respect and comfort in primary healthcare encounters.

The tendency of female participants towards same-gender physicians highlights the lasting impact of cultural and religious norms on patient comfort and openness in Saudi primary healthcare. This insight adds to wider conversations about the ways in which culturally aware staffing policies and training can improve patient-centered care in conservative environments.

This study provides valuable, context-specific insights into the manifestation of factors influencing patient satisfaction within Saudi primary health care (PHC), aligning with findings from international literature. The findings indicate that although patient-centered care principles are gaining recognition in policy documents, a significant number of primary care providers remain unfamiliar with operational concepts like patient enablement and empowerment. The concepts central to the patient-centered model have not yet been fully integrated into primary healthcare practice or undergraduate medical curricula in Saudi Arabia. The qualitative accounts provided here offer a comprehensive understanding of the cultural and systemic factors that contribute to the challenges of implementing patient-centered care. Hierarchical relationships between physicians and patients, time limitations, and insufficient communication training all impede empathic engagement and collaborative decision-making.

To attain authentic transformation, medical education and ongoing development must focus on empathy, active listening, collaborative decision-making, and empowering patients. Incorporating these values into clinical guidelines, performance evaluations, and primary health care reform initiatives has the potential to connect policy aspirations with practical implementation. This study enhances the understanding of how Saudi primary healthcare can progress towards more holistic and empowering care by contextualizing global patient-centered principles within local cultural and institutional realities.

### Limitations

This research presents a number of limitations. First, the collection of qualitative data through semi-structured interviews carried out by one of the researchers may have been subject to interviewer bias, potentially affecting both the manner in which questions were posed and the responses provided by participants. Reflexive journaling and peer debriefing were employed to reduce this risk and encourage neutrality. Second, the research was carried out at a single primary health care center, which could restrict the applicability of the findings to other areas or healthcare environments in Saudi Arabia. Future research may involve multiple locations or a comparison between urban and rural primary health care centers to improve generalizability. Third, although the study offers valuable insights derived from patient interviews, it did not employ methodological triangulation (such as integrating patient, provider, and document data); the inclusion of diverse data sources in future research could enhance the thoroughness and reliability of the findings. Fourth, the study used a qualitative method, considering it was exploratory in nature. Contextual bias and problems with data processing and presentation are all features of qualitative research (76). However, the experience and diversity of the team involved in this research, along with a clear description of the methods included, contributed to alleviating these issues. Furthermore, it is essential to recognize potential response biases. Participants might have been affected by the Hawthorne effect, leading them to offer more positive descriptions of their experiences due to their awareness of being involved in a research study. Similarly, the influence of social desirability bias may have impacted the extent to which participants candidly shared negative experiences or critiques regarding their primary care physicians. To mitigate these effects, the interviewer underscored the importance of confidentiality, employed neutral and open-ended questions, and fostered a comfortable environment to promote candid responses. However, it is important to acknowledge that these biases cannot be completely eliminated. Finally, the online format of the interviews may have resulted in the underrepresentation of individuals who do not have access to reliable internet or digital devices. This may have resulted in a sample that is biased towards participants with elevated educational or socioeconomic backgrounds, which could affect the range of perspectives represented. Future research should investigate if the relationship we observed varies based on specific patient characteristics. Moreover, some participants may be incapable or disinclined to articulate their experience during the interview. Investigating the impact of cultural and other factors on the articulation of patient satisfaction during consultations would be highly beneficial for future research. Finally, as this study concentrated on individuals with chronic diseases under the care of a primary care physician, the results may not apply to others with more acute needs. One strength of our study is the relatively large sample size in this qualitative research (*n* = 46). The interviews offered a chance for patients receiving usual care to share valuable insights regarding the care delivered by their PCPs in managing their chronic diseases. Future research could also utilize comparative or longitudinal qualitative designs to investigate patient satisfaction across various regions and healthcare settings in Saudi Arabia. Analyzing urban and rural primary health care centers, as well as public and private facilities, would provide insights into the differences in accessibility, communication, and cultural expectations.

## Conclusion

The findings highlight the significance of enhancing communication that prioritizes patients and providing care that is sensitive to cultural contexts in Saudi primary healthcare. Training programs for primary care physicians must focus on active listening, empathy, and honoring patients’ preferences, including considerations of gender and privacy. At the system level, improving coordination between primary health care and specialty services, enhancing continuity of care, and incorporating mental health support into primary health care can lead to better patient satisfaction and health outcomes.

Future studies might utilize comparative or longitudinal qualitative designs across various Saudi regions or healthcare settings to explore differences in patient experience. Incorporating a diverse range of stakeholders, patients, providers, and administrators, into research efforts would yield a more thorough understanding of the factors influencing satisfaction and inform evidence-based reforms in primary health care.

Additionally, this study examined patients under the care of primary care physicians associated with hospital-based primary health care centers. This offered important insights into structured primary healthcare delivery; however, patient experiences may vary among those treated by community-based or private primary care physicians who function outside hospital networks. Future research may consider employing a comparative qualitative design to investigate these variations, focusing on the impact of institutional settings, resources, and organizational culture on patient satisfaction and the dynamics of patient–physician relationships. These comparisons would offer a deeper insight into the dynamics of primary care throughout Saudi Arabia.

## Data Availability

The raw data supporting the conclusions of this article will be made available by the authors, without undue reservation.
